# Microbial Community Succession and Its Environment Driving Factors During Initial Fermentation of Maotai-Flavor Baijiu

**DOI:** 10.3389/fmicb.2021.669201

**Published:** 2021-05-06

**Authors:** Fei Hao, Yuwei Tan, Xibin Lv, Liangqiang Chen, Fan Yang, Heyu Wang, Hai Du, Li Wang, Yan Xu

**Affiliations:** ^1^Kweichow Moutai Distillery Co., Ltd., Guizhou, China; ^2^Key Laboratory of Industrial Biotechnology, Center for Brewing Science and Enzyme Technology, Ministry of Education, Jiangnan University, Wuxi, China; ^3^Kweichow Moutai Group, Guizhou, China

**Keywords:** Maotai-flavor Baijiu, high throughput sequencing, microbiol community succession, solid-state fermentation, environmental driving forces

## Abstract

The microbial composition and environmental factors can take a great influence on community succession during the solid-state fermentation (SSF) of Maotai-flavor Baijiu. In this paper, high-throughput sequencing was used to reveal the dominant microorganisms and the evolution process of microbial community structure in the initial fermentation of Maotai-flavor Baijiu. The correlation analysis was carried out for the relationship between physicochemical factors and fermented microbes. The results showed that microorganisms were obviously enriched and the diversity of bacteria and fungi showed a downward trend during the heap fermentation process of Maotai-flavor Baijiu. However, the diversity of fungi in the pit fermentation process increased. Generally, *Lactobacillus*, *Pichia*, and *Saccharomyces* were the dominant microorganisms in the initial fermentation of Maotai-flavor Baijiu. According to the redundancy analysis, we found that reducing sugar was the key driving factor for microbial succession in the heap fermentation, while acidity, alcohol, and temperature were the main driving forces in pit fermentation. This study revealed the microbial succession and its related environmental factors in the initial fermentation of Maotai-flavor Baijiu, which will enrich our knowledge of the mechanism of solid-state liquor fermentation.

## Introduction

Baijiu is produced by the solid-state fermentation (SSF) process which involves complex microbiota (Wang et al., 2018a; Guo et al., 2019). Complex microbial succession during SSF plays an important role in yield and quality of liquor production (Li et al., 2016a; Liu et al., 2017; Zhang et al., 2020). The multiple environmental factors drive microbial community changes at large spatial scales, including pH, temperature, moisture, and salinity (Zheng et al., 2014a; Xu et al., 2018). Understanding the relationship between microbial community and environment factors in fermentation process is helpful to provide controllable management strategies.

Recently, the research of Baijiu technology mainly focused on the screening of functional microorganisms, analysis of microbial community structure succession, and the correlation between volatile profiles and microbial communities (Zou et al., 2018; Jin et al., 2019; Song et al., 2019; Wang et al., 2020). These studies revealed the changes of microbial succession in the process of liquor making, the core functional strains for liquor brewing, and their contribution to liquor flavor compounds. That is a great significance for the analysis of microbial brewing mechanism. However, little is known about the relationship between environmental factors and microbial structure succession, which makes it difficult to control and manage the fermentation process. In addition, the production process of liquor is accompanied by special extreme environments, such as high temperature, high acidity, and high ethanol (Xu et al., 2017; Wang et al., 2019a). That deepens the difficulties to study the microbial community diversity and temporal succession in microbial ecology of Maotai-flavor Baijiu fermentation.

Maotai is a world-famous traditional Baijiu with complex taste and aroma, which are considered to be strongly influenced by the quality of fermentation technology (Xu and Ji, 2012). Maotai production is carried out in nine batches over the course of a year. Two batches of sorghum are added to the liquor brewing process during the first two batches, which is called the initial fermentation of Maotai flavor Baijiu. Sorghum is gelatinized after absorbing water, and then produced various flavor substances and their precursors, which laid a foundation for the smooth progress of subsequent liquor-making process. However, the core microorganisms and the environmental driving factors of community succession are not clear.

In this paper, the microbial composition during the initial fermentation of Maotai-flavor Baijiu was analyzed by high-throughput sequencing technology. The correlation between the microbial composition and the changes of physicochemical indexes was analyzed using redundancy analysis. This work aimed to explore the environmental driving force of microbial succession.

## Materials and Methods

### Sample Collection

All samples were collected in a well-known sauce-flavor *Baijiu* distillery (Guizhou Province, China). The fermentation process was carried out in two distinct phases: heap fermentation and pit fermentation. Heap fermentation samples were collected on days 0, 1, 2, and 3, marked as D0, D1, D2, and D3, respectively (Supplementary Figure S1). For pit fermentation, samples were collected at 5-day intervals until the end of fermentation and marked as F0, F5, F10, F15, and F30 (Supplementary Figure S1, point C and D for heap fermentation samples, point A and B for pit fermentation samples). Samples were taken from two layers for replicate samples, and different points in the same layer were mixed to form one sample to reduce the heterogeneity of samples before extraction and analysis (Song et al., 2017). Hence, we totally collected eight samples of heap fermented grains and 10 samples of pit fermented grains.

### Fermentation Parameters Detection and Analysis

To understand the fermentation processes, seven fermentation parameters, including temperature, moisture, acidity, reducing sugar, alcohol, acetic acid, and lactic acid were detected. The temperatures of the sampling locations were measured and recorded by electron probe thermometer before sample collection. Moisture of fermented grains was determined by a gravimetric method by drying samples to a constant weight at 125°C. The acidity was measured based on the methods described by others (Tan et al., 2019). Alcohol, reducing sugar, and organic acids were analyzed *via* high-performance liquid chromatography (HPLC; Waters 2,695, Milford, United States) equipped with refractive index detector (RID, 2414) and photodiode array detector (PDA, 2998), based on the method described elsewhere (Zheng et al., 2014b; Wang et al., 2017; Jiang et al., 2019).

### DNA Extraction, Amplicon Sequencing, and Analysis

Sample pretreatment and DNA extraction were using a previously reported method (Du et al., 2019). Bacterial V3-V4 and the fungal ITS1 of the rRNA were amplified using forward primers (5'-GTACTCCTACGGGAGGCAGCA-3', 5'-CTTGGT CATTTAGAGGAAGTAA-3') and the reverse primer (5'-GT GGACTACHVGGGTWTCTAAT-3', 5'-TGCGTTCTTCATCGATGC-3'), respectively (Soergel et al., 2012; Hertz et al., 2016). The barcoded PCR products were sequenced on a MiSeq benchtop sequencer for 250-bp paired-end sequencing (2 × 250 bp; Illumina, San Diego, CA, United States) at Beijing Auwigene Tech. Ltd (Beijing, China). All the raw sequences generated were processed *via* QIIME v.1.9.1 (Caporaso et al., 2010) and R (v.2.3–5). The representative bacterial OTU sequences were annotated using the Silva 132_16 S rRNA database with a QIIME-based wrapper of RDP-classifier (v.2.2). The representative fungal OTU sequences were compared using BLAST search against the UNITE fungal ITS database.^1^ All sequences generated were submitted to the NCBI database under accession number: PRJNA702253.

### Statistical Analysis

The dynamics of fermentation parameters and microbial diversity were fitted *via* Excel2019 (Microsoft Corporation, United States). To analyze the community driving factors, distance-based redundancy analysis (db-RDA) was conducted *via* Canoco software. The Mantel test was conducted in R (version 3.2.4) *via* the vegan package (version 2.3–4). All possible Spearman’s rank correlations among the genera and fermentation parameters using R (v.2.3–5) and the significant correlations (FDR < 0.05) were retained. The network was created by Gephi (v 0.9.2) to sort through and visualize the correlations between microbiota and fermentation parameters (Kauffman et al., 2014).

## Results

### Dynamic Changes of Fermentation Parameters During Initial Fermentation of Maotai-Flavor Baijiu

**Figure 1:**
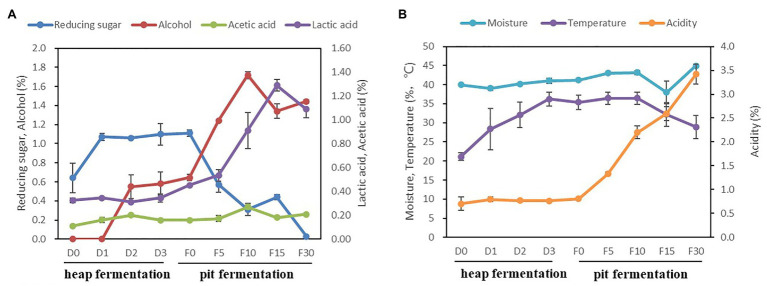
Changes of physicochemical parameters in fermented grains during fermentation. **(A)** The trend of reducing sugar, alcohol, acetic acid, and lactic acid. **(B)** The trend of moisture, temperature, and acidity.

The seven fermentation parameters, including temperature, moisture, acidity, reducing sugar, alcohol, acetic acid, and lactic acid were detected and analyzed during Baijiu fermentation (Figure 1). In the stage of heap fermentation, alcohol increased to 0.58 ± 0.12% at the 3rd day. Reducing sugar increased significantly from day 0 to 3, with an average range from 0.64 ± 0.16% to 1.06 ± 0.01%, while the temperature increased from 21.2 ± 1.0°C to 36.2 ± 1.8°C. The acidity, lactic acid, acetic acid, and moisture also showed an upward trend. In the pit fermentation, reducing sugar and temperature decreased from 1.11 ± 0.04%, 35.4 ± 2.0°C to 0.20 ± 0.03%, 28.9 ± 3.1°C, respectively. The values of alcohol, acidity, lactic acid, and acetic acid increased significantly, from 0.64 ± 0.03%, 0.80 ± 0.01%, 0.45 ± 0.01%, and 0.16 ± 0.004% to 1.44 ± 0.01%, 3.42 ± 0.01%, 1.09 ± 0.07%, and 0.21 ± 0.01%, respectively.

### Microbial Community Composition and Diversity During Initial Fermentation of Maotai-Flavor Baijiu

**Figure 2:**
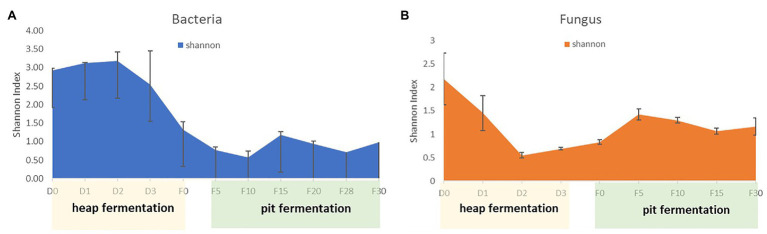
Diversity of microbiol community in fermented grains during fermentation. **(A)** Diversity of bacterial community in fermented grains during fermentation. **(B)** Diversity of fungal community in fermented grains during fermentation.

The high-throughput sequencing was applied to reveal the microbial community structure of samples. After quality control, 553,936 high quality reads from V3 to V4 region of 16S rRNA gene sequences, and 487,046 high quality reads from ITS region were obtained from 18 samples. For bacteria, there was an average of 30,774 reads per sample, with a range from 20,066 to 69,274 reads. For fungi, there was an average of 27,058 reads per sample, with a range from 20,036 to 53,039 reads. Bacterial and fungal OTUs were clustered at a 97% similarity level of sequences. Measures of α-diversity revealed the different trends between bacterial and fungal at different fermentation stages (Figure 2). The diversity of bacterial community increased in the early stage of heap fermentation, and decreased continuously from the late stage of heap fermentation to the early stage of pit fermentation. And then it was increased slightly and kept stable in the late stage of pit fermentation. However, the diversity of fungal community decreased in the early stage of heap fermentation and increased continuously from the late stage of heap fermentation to the early stage of pit fermentation. And then it was decreased slightly and kept stable in the process of pit fermentation.

**Figure 3:**
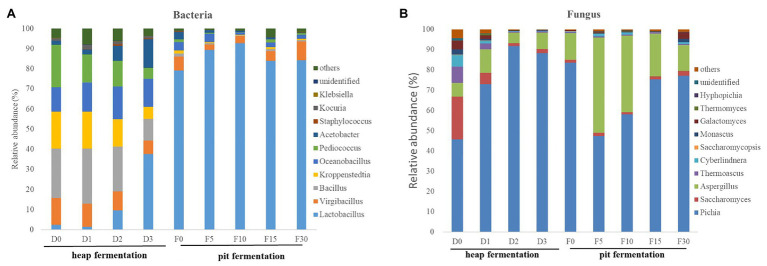
Relative abundance of microbiol community in fermenting grains sampled from different fermentation stages. **(A)** Average bacterial distribution at the genus-level of microbiota. **(B)** Average fungal distribution at the genus-level of microbiota.

A total of 210 genera of bacteria and 75 genera of fungi were detected in fermented grains of Maotai-flavor Baijiu. The genera with an average relative abundance over 0.1% across all the samples were selected. In particular, *Lactobacillus* (59.6%), *Virgibacillus* (8.2%), unidentified (7.4%), *Kroppenstedtia* (6.8%), *Bacillus* (5.7%), *Oceanobacillus* (5.4%), and *Pediococcus* (3.1%) were the dominant genera. In the process of heap fermentation, the relative abundance of *Lactobacillus*, *Kroppenstedtia*, and *Pediococcus* increased gradually, while *Virgibacillus*, *Bacillus*, and *Oceanobacillus* decreased. And in pit fermentation, *Lactobacillus* increased continuously and became the absolute dominant bacteria (Figure 3A).

The main fungal genera (with an average abundance above 1%) in fermented system were *Pichia* (71.1%), *Saccharomyces* (18.2%), unidentified (4.2%), *Aspergillus* (1.6%), *Thermoascus* (1.3%), and *Monascus* (1.2%). In heap fermentation, the proportion of *Pichia* was increasing and became the dominant bacterial genus (Figure 3B), while the other major fungal genera continues to decrease. In pit fermentation, although the abundance of *Pichia* has decreased, it was still the major fungal genus with the highest proportion. In addition, the abundance of *Saccharomyces* has been increasing and also becoming one of the main fungal microorganisms.

### Driving Factors of Heap Fermentation and Pit Fermentation Process

**Figure 4:**
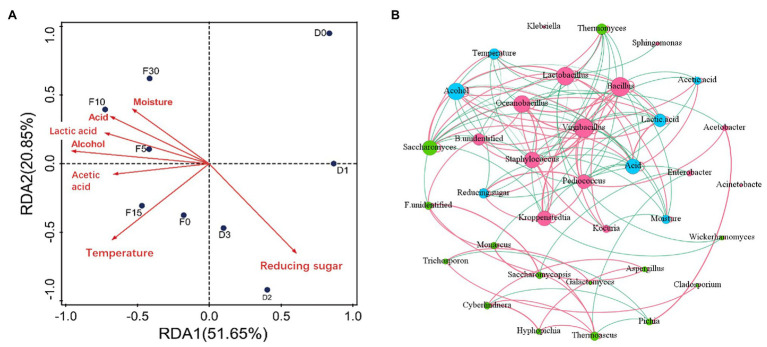
Correlations between fermentation parameters and community composition during heap fermentation and pit fermentation stages. **(A)** Distance-based-redundancy analysis (RDA)-forwsel analysis of microbial community composition and physicochemical characteristics. The last capital letter D and F represents heap fermentation and pit fermentation, respectively. The numbers after the letters represent the fermentation days of 0, 1, 2, 3, 5, 10, 15, and 30. The significance of variables was assessed with the permutation test (*n* = 1,000). **(B)** Co-occurrence network of physicochemical properties and microbes based on the spearman correlation. Red, green, and blue circles represent bacteria, fungi, and physicochemical properties, respectively. Red gray and green gray represent positive and negative interactions, respectively.

The physicochemical parameters in fermentation can affect the growth and metabolism of microorganisms. It is helpful to understand the mechanism of fermentation process by analyzing the correlation between microorganisms and environmental factors. To clarify the main drivers in different fermentation process, distance-based redundancy analysis (db-RDA) and correlation network between microbiota and driving factors were performed as shown in Figure 4A. Results of db-RDA analyses showed that the explanatory rate of physicochemical indices of samples on the distribution of microbial communities was 72.4%. That indicated that physicochemical indices had an important impact on microbial succession change. Besides, reducing sugar, temperature, alcohol, and acidity had strong correlation with microbial community. Among them reducing sugar in heap fermentation was positively correlated with microbial community. Reducing sugar had higher content in this stage. Adequate sugar supply is benefited for microbial growth and metabolism (Chen et al., 2014). Sugar content was the main driving factor for microbial succession in this stage. Ethanol, acidity, temperature, and other indicators were positively correlated with the microbial community during pit fermentation process. In the pit fermentation period, microorganisms metabolize to produce alcohol and lactic acid (Yi et al., 2019). Ethanol, acidity, and temperature were the main driving factors for microbial succession in the process of pit fermentation.

To clarify the relationships among specific genera and driving forces, we analyzed their correlation *via* Spearman coefficient (*p* < 0.05; Figure 4B). From a network perspective, Figure 4B shows that the genera *Virgibacillus*, *Kroppenstedtia*, and *Pediococcus* were positively correlated with sugar content. The result indicated that there was a certain correlation between sugar content and the dominant bacteria in the process of heap fermentation. Ethanol, acidity, lactic acid, and temperature were positively correlated with *Lactobacillus* and *Saccharomyces*. This indicated that the above physicochemical parameters had an important impact on the dominant microorganism succession in pit fermentation. We also found that acetic acid was negatively correlated with *Virgibacillus* and *Bacillus*, and water was negatively correlated with *Virgibacillus*, *Bacillus*, *Oceanobacillus*, *Staphylococcus*, and *Kocuria*.

## Discussion

Baijiu fermentation is an open SSF process, in which microorganisms are derived from the environment, and the environmental factors affect the succession of microbial community structure (Guan et al., 2020; Wang et al., 2020). However, there are few studies on the interactions of microbial and environmental factors in Maotai-flavor Baijiu fermentation process; the lack of knowledge makes it difficult to control the fermentation process. Therefore, it is important to determine the mechanisms underlying the assembly and succession of the heap fermentation and pit fermentation microbial community.

The traditional Maotai-flavor Baijiu fermentation is processed by typical two-stage fermentation, including heap fermentation and pit fermentation. Heap fermentation is a special brewing process of Maotai-flavor Baijiu, which is different from other distilled liquors in the world (Tsakiris et al., 2014; Wiśniewska et al., 2017). Due to the growth and metabolism of microorganisms, the temperature of fermented grains in the process of heap fermentation can reach above 50°C. Heap fermentation enriches and selects appropriate microorganisms for liquor fermentation. And these selected microbiota produce various flavors and flavor precursors, which are the key to the Maotai flavor (Wang et al., 2018b; Dai et al., 2020). In this study, the microbial succession in the heap fermentation was investigated, and the results showed that the main genera were *Virgibacillus*, *Kroppenstedtia*, *Bacillus*, *Lactobacillus*, *Pichia*, *Saccharomyces*, and *Thermoascus* (Figure 3). The dominant microorganisms in heap fermentation process are heat-resistant species, which is consistent with the previous research results (Wu and Xu, 2012).

During the heap fermentation process, the diversity of bacteria and fungi showed the opposite trend. In the beginning of heap fermentation, the stater of Baijiu fermentation, which was called daqu, was added to the fermented grains. Bacteria were the dominant microorganisms in daqu (Gan et al., 2019), which led to the growth of bacteria community in the early stage of heap fermentation. However, in the late stage of heap fermentation, the amount of fungal community increased (Wu et al., 2012), and the diversity of fungi showed an increasing trend. It is noteworthy that the reducing sugar content kept stable in the late stage of heap fermentation; however, the reducing sugar in heap fermentation was positively correlated with microbial community. Baijiu production process is solid state fermentation, saccharification and fermentation were carried out simultaneously. Therefore, the content of reducing sugar in fermented grains is a dynamic parameter, and the content of reducing sugar remains stable in the process of heap fermentation, indicating that the production and consumption of reducing sugar reach a balance. Microorganisms decomposed the fermented grains to produce reducing sugars, so the reducing sugars could be used as nutrients for their growth and metabolization. Our results show that the heap fermentation enriched the necessary microflora for the production of Maotai-flavor Baijiu.

The pit fermentation is the main stage of alcohol accumulation in Maotai-flavor liquor production. During the pit fermentation process, *Lactobacillus* became the dominant bacteria. Meanwhile, the abundance of *Saccharomyces* increased significantly, and together with *Pichia* it has become the dominant fungi in the fermentation system. Compared with previous studies (Song et al., 2017), we found that the content of ethanol and lactic acid increased significantly during the pit fermentation process, while the temperature gradually decreased in the later stage of pit fermentation (Figure 1). It implied the flourishing growth of the microbial community with specific functional metabolites produced (Li et al., 2016b; Kong et al., 2017). With the content of ethanol and lactic acid increased, the extreme environmental factors inhibited the growth of most bacteria, and consequently, the diversity of bacteria decreased. However, some fungi (such as *Saccharomyces* and *Saccharomycopsis*) still maintained a good growth trend, which led to the increasing of fungal diversity in the early stage of pit fermentation process. At the end of pit fermentation, the fermented grains were got out from the pit for the spirit distillation, which led to the anaerobic fermentation environment is interrupted. Part of the alcohol and lactate could be converted into esters and other flavor substances in the late stage of pit fermentation (Jia et al., 2020), so it showed a decreasing trend of the alcohol and lactate content. These results showed that the key functional fermentation microorganisms could be enriched in pit fermentation process, which provided the basis for the continuous fermentation of Maotai-flavor Baijiu.

With the successive changes of microbial communities in the Maotai-flavor Baijiu fermentation process, *Lactobacillus*, *Saccharomyces*, and *Pichia* became the dominant microorganisms in the fermentation system (Figure 3). These microorganisms are common to different flavor liquor brewing processes. However, they show different succession trends in different brewing systems due to differences in raw materials, environment, and process operation (Tan et al., 2019; Wang et al., 2019b; Pang et al., 2020). *Lactobacillus* can regulate the acidity of fermented grains by lactic acid metabolism and inhibit the growth of contaminating bacteria (Du et al., 2020). *Lactobacillus*, as the core functional microorganism for the increasing acidity, has ability to produce lactic acid, ethanol, and acetic acid by heterolactic fermentation (Song et al., 2017). *Saccharomyces* has acid resistance and mainly metabolizes ethanol during fermentation in pits (Lu et al., 2015; Meng et al., 2015). It plays an important role in diversity of tastes and flavors with established qualities (Abe et al., 2019). *Pichia* was considered as non-alcoholic yeast during the liquor making process, mainly used to produce volatile compounds in liquor (Mónaco et al., 2016; Hu et al., 2018). The previous research found that *Pichia* could degrade lactic acid and upregulate the microbial metabolic activity of ethanol in *S. cerevisiae* under lactic acid stress (Mónaco et al., 2014; Yamamoto et al., 2019; Deng et al., 2020). It indicated that *Pichia* could be an important acidity-regulating microorganism for the production of liquor-making. Accordingly, the process of heap and pit fermentation significantly contributed to the enrichment of microorganisms, thus, forming the unique flavor of Maotai-flavor Baijiu. The brewing process of Maotai-flavor Baijiu is a complex microecological fermentation system, and the dynamic changes of microbial succession in this process need to be further studied.

In conclusion, we unveiled the microbial diversity and composition during the initial fermentation of Maotai-flavor Baijiu by high-throughput sequencing technologies. Both heap fermentation and pit fermentation play an important role in the screening and enrichment of microbial community for Maotai-flavor Baijiu production. In addition, reducing sugar was the key driving factor for microbial succession in the heap fermentation, while acidity, alcohol, and temperature were the main driving forces in pit fermentation. According to the best of our knowledge, this is the first report to analyze the dynamic changes of microorganism in the initial fermentation of Maotai-flavor Baijiu. Exploring the microbial succession and its relative environmental factors could provide valuable information for understanding the complete ecology of Maotai-flavor Baijiu fermentation systems.

## Data Availability Statement

The datasets presented in this study can be found in online repositories. The names of the repository/repositories and accession number(s) can be found in the article/Supplementary Material.

## Author Contributions

FH and YT are responsible for sample collection, gene extraction, and data analysis. XL and LC are responsible for sample collection and gene extraction. FY, HW, and HD are responsible for the design of specific ideas and technical guidance of this study. LW and YX are responsible for the overall design, organization, and implementation of this study. All authors contributed to the article and approved the submitted version.

### Conflict of Interest

LW was employed by Kweichow Moutai Group. FH, XL, LC, FY, and HW were all employed by Kweichow Moutai Distillery Co., Ltd.

The remaining authors declare that the research was conducted in the absence of any commercial or financial relationships that could be construed as a potential conflict of interest.

## Footnotes

^1^https://unite.ut.ee/
