# Supplementary material for: Microbial Community Succession and Its Environment Driving Factors During Initial Fermentation of Maotai-Flavor Baijiu
Source: Front Microbiol. 2021 May 6;12:669201. doi: 10.3389/fmicb.2021.669201 (PMC8139626; doi:10.3389/fmicb.2021.669201)
Supplement: Supplementary file 1 [file Table_1.DOCX]

Supplementary Material

## Supplementary Figures


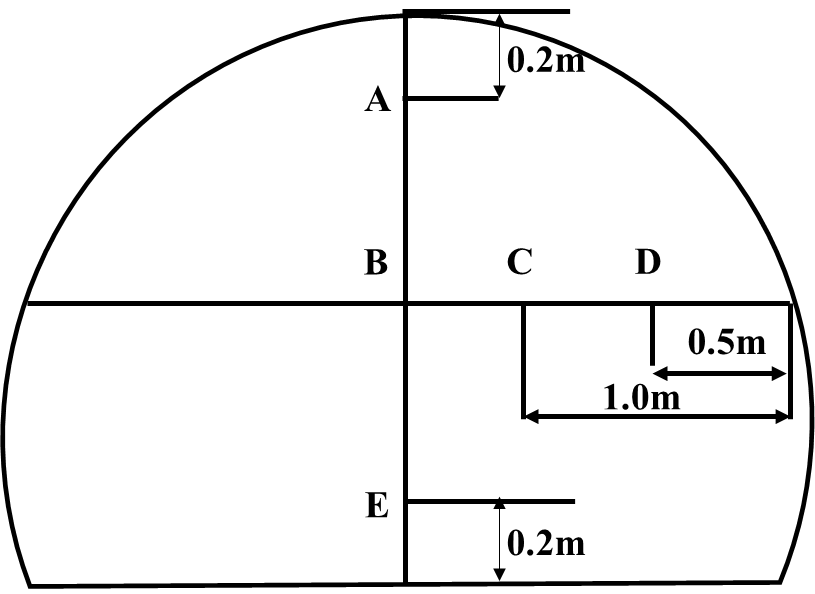

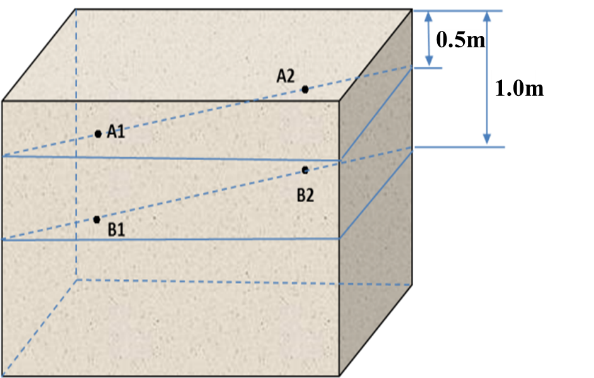


**Supplementary Figure 1. Sampling points of fermented grains during the solid state fermentation. (A) heap fermentation, (B) pit fermentation.**
